# Receptor tyrosine kinases and downstream pathways as druggable targets for cancer treatment: the current arsenal of inhibitors

**DOI:** 10.1186/s12943-018-0792-2

**Published:** 2018-02-19

**Authors:** Wagner Ricardo Montor, Andrei Ronaldo Oliveira Silva Escartin Salas, Fabiana Henriques Machado de Melo

**Affiliations:** Departamento de Ciências Fisiológicas, Faculdade de Ciências Médicas da Santa Casa de São Paulo, São Paulo, Brazil

**Keywords:** Receptor tyrosine kinases, tyrosine kinase inhibitors, MAPK signaling pathways, *RAS*-mutations, BRAF driven cancers, PI3K/AKT transduction network

## Abstract

Searching for targets that allow pharmacological inhibition of cell proliferation in over-proliferative states, such as cancer, leads us to finely understand the complex mechanisms orchestrating the perfect control of mitosis number, frequency and pace as well as the molecular arrangements that induce cells to enter functional quiescence and brings them back to cycling in specific conditions. Although the mechanisms regulating cell proliferation have been described several years ago, never before has so much light been shed over this machinery as during the last decade when therapy targets have been explored and molecules, either synthetic or in the form of antibodies with the potential of becoming cancer drugs were produced and adjusted for specific binding and function. Proteins containing tyrosine kinase domains, either membrane receptors or cytoplasmic molecules, plus the ones activated by those in downstream pathways, having tyrosine kinase domains or not, such as RAS which is a GTPase and serine/threonine kinases such as RAF, play crucial role in conducting proliferation information from cell surroundings to the nucleus where gene expression takes place. Tyrosine kinases phosphorylate tyrosine residues in an activating mode and are found in important growth factor receptors, such as for ligands from families collectively known as VEGF, PDGF and EGF, to name a few and in intracellular downstream molecules. They all play important roles in normal physiology and are commonly found mutated or overexpressed in neoplastic states. Our objective here is to present such kinases as druggable targets for cancer therapy, highlighting the ones for which the pharmacological arsenal is available, discussing specificity, resistance mechanisms and treatment alternatives in cases of resistance, plus listing potential targets that have not been successfully worked yet.

## Background

Cells communicate with the microenvironment through several ways and the membrane bound receptors which can be triggered by specific ligands are undoubtedly one of the most important communication pathways. Ligand receptor stimulation is involved in several cell mechanisms, such as control of cell proliferation, migration, differentiation, apoptosis and others. Tumor cells proliferate faster or proliferate when a quiescent state would be desirable and they do so because there are excess growth factors in the microenvironment, there are more membrane bound receptors, or these receptors or downstream signaling pathways are constantly activated by mutations or chromosome rearrangements. Here we discuss a specific family of such receptors and downstream signaling molecules, the tyrosine kinase receptors and the cytoplasmic molecules they activate, some of them, such as RAF being serine/threonine kinases but directly activated in tyrosine kinase receptor pathways, their role in normal cell proliferation and their role as targets for molecules designed to control cell proliferation in cancer.

## Receptor tyrosine kinases

### Epidermal Growth Factor Receptors (EGFR)

As will be described below, receptor tyrosine kinases (RTKs), being membrane anchored, indirectly send signals to the cell nucleus through cytoplasmic pathways involving a series of molecules that eventually culminate with translocation of specific proteins from the cytoplasm activating and/or acting as transcription factors orchestrating proliferation through gene expression [1].

One of the most important receptor tyrosine kinases to have a role in cancer cell proliferation is EGFR, the epidermal growth factor receptor, a transmembrane glycoprotein member of the ERBB receptor tyrosine kinase superfamily leading to a phosphorylation cascade mediated via tyrosine kinases which works downstream through the PI3K–PTEN–AKT, MAPK, ERK, and JAK/STAT pathways and promotes proliferation, invasion, angiogenesis, and metastatic spread. EGFR expression is found to be altered or the receptor is found to be mutated in several types of cancer, including lung, breast, head and neck and gastrointestinal tumors for example [2, 3].

For some of these tumors it is standard of care to test for EGFR expression and mutations in order to define pharmacological management with EGFR inhibitors, which can be either small molecules known as tyrosine kinase inhibitors (TKIs) or monoclonal antibodies [3, 4]. *EGFR* mutations play an important role in lung cancer and the most common ones found in non-small cell lung carcinoma (NSCLC), for example are short in frame deletions in exon 19 and the point mutation L858R in exon 21 [2, 3]. Tumors harboring these DNA alterations are sensitive to specific TKIs such as gefitinib and erlotinib, known as first generation TKIs for EGFR inhibition, prolonging patient’s progression-free survival (PFS) in several months when compared to chemotherapy alone [5–7]. Although about 15% of NSCLC patients present mutations in EGFR making them eligible for TKI treatment, resistance to these drugs is commonly seen in about one year of treatment and that is mostly due to a secondary T790M mutation in exon 20, other than alternative pathway activation. Second generation *EGFR* TKIs such as afatinib circumvented resistance elicited by T790M mutation providing improved PFS and Overall Survival (OS) [5, 8], but more encouraging are the third generation EGFR TKIs of which the most successful example is osimertinib, fully approved by the Food and Drug Administration (FDA) and European Comission for treating cancers that harbor the EGFR T790M mutation. Osimertinib is a mono-anilino-pyrimidine compound that irreversibly binds with cysteine residue in position 797 of mutant EGFR while having little effect on wild type EGFR. Other third generation EGFR TKIs include rociletinib and olmutinib but the development of those did not advance as osimertinib due to emergence of severe adverse effects [9–12].

Besides promising and effective, the treatment with third generation EGFR TKIs showed that resistance can still reemerge, due to further modifications in the receptor, mainly C797S mutation but also alternative pathway activation, such as those involving *HER2* and *MET* amplification or G12S *KRAS* mutation, other than histologic transformation in the case of NSCLC, making them phenotypically transform into small cell lung cancer (SCLC) [13, 14]. In order to circumvent third generation EGFR TKI resistance, screening a library of about 2.5 million compounds, EAI045 was found to overcome T790M and C797S mediated resistance being an allosteric inhibitor of EGFR, promoting tumor shrinkage in combination with cetuximab in mice tumors harboring L858R, T790M and C797S mutations. Although promising, laboratory adjustments and clinical trials are still needed for this compound [15].

Variants of the EGFR family play important roles in other tumors, such as breast cancer. EGFR is a family of receptors that act dimerizing on cell membranes through the combination of four specific family members, namely HER-1, HER-2, HER-3 and HER-4. HER-2 is overexpressed in about 20% of breast cancers, against which a monoclonal antibody called trastuzumab has been developed. Small molecule TKIs such as lapatinib also target HER-2 and an open-label, multicenter, phase III study showed benefits of the combined use of lapatinib and trastuzumab compared to single HER-2 inhibition which can be explained by the fact that these two molecules inhibit HER-2 in distinct and complementary ways, trastuzumab being specific for the non-activated receptor and lapatinib being specific to the ligand-bound receptor. As lapatinib increases HER-2 in the membrane and trastuzumab triggers antibody mediated cellular cytotoxicity, their combination improved response comparing to single inhibition [16]. Following the same pattern of combined therapy for potentializing results, the inclusion of pertuzumab, another monoclonal antibody targeting HER-2, but specifically inhibiting HER-2/HER-3 heterodimer formation showed improved OS in a randomized phase III trial, in comparison to conventional treatment [17].

As different tumors are molecularly characterized, the contribution of HER-2 overexpression to tumorigenesis and tumor progression becomes more evident and new existing therapeutic approaches can then be tested. A recent phase II trial evaluated the effect of afatinib in HER-2 positive platinum resistant urothelial carcinomas. The overall response rate (ORR) was 8,6% and not enough number of patients benefited in order to enroll more patients but it is a pathway of exploration for new approaches that can eventually lead to good results [18] as seen for HER-2 positive gastric cancers in which OS was improved by trastuzumab when compared to chemotherapy alone [19]. The scenery of mutations found in tumors is vast and difficult to fully characterize in the clinical setting, as it is difficult to understand and justify why specific tumors express specific receptors, such as breast cancer cells and HER-2, but since the arsenal of pharmacological options is developed, targets for these molecules are searched in tumors, opening doors for new therapies.

Still in the list of specific EGFR inhibitors we find cetuximab and panitumumab, FDA approved monoclonal antibodies used mainly but not only in metastatic colorectal, head and neck and NSCLC when non-mutated *KRAS* is present. As KRAS is a downstream molecule in EGFR signaling, its mutation abrogates any benefit from EGFR inhibition [20] and attempts to make KRAS druggable are presented elsewhere in this text.

### Platelet Derived Growth Factor Receptor (PDGFR)

Another tyrosine kinase growth factor receptor family that regulates cell division is the PDGFR, platelet-derived growth factor receptor. The receptors act as homo or heterodimers of the AA, BB or AB type, being present in a wide range of cells. The intracellular domain of the receptor presents tyrosine residues that can be autophosphorylated upon receptor activation and this way serve as binding sites for SH2 containing proteins which can be enzymes or non-enzymatic molecules. Overall, the activation of this receptor orchestrates a rearrangement of molecules within the cytoplasm, through approximation of potential interactors and that leads to downstream cascades promoting proliferation [21].

The incidence of activating defects in PDGFR in cancer is about 30% and that includes mutations, deletions and amplification, according to studies found in The Cancer Genome Atlas (TCGA). Considering tumor types in which PDGFR is altered in at least 10% of the cases we find melanoma, lung cancer, glioblastoma, bladder, prostate, colorectal and ovarian cancers [21].

Small molecules targeting this receptor have been developed, imatinib being the first one to be used in the clinical setting. Imatinib revolutionized the treatment of chronic myeloid leukemia (CML) in 2001, a disease in which the *BCR-ABL* fusion occurs. The BCR-ABL fusion protein is inhibited by PDGFR inhibitors and vice-versa, because of their similar structure, so imatinib and the most modern related TKIs such as dasatinib, nilotinib and ponatinib will be described below, in the ABL1 section.

A very low percentage of C-KIT negative gastrointestinal stromal tumors (GIST) contain *PDGFRA* mutations, benefiting from imatinib in a way comparable to CML patients do, although C-KIT positive GIST also respond not only to imatinib but also sunitinib, eventually developing resistance. A phase III trial showed that patients who develop resistance to both imatinib and sunitinib, evolving to fatal disease can still respond to the less specific TKI regorafenib when compared to the placebo [22]. The same way a rare condition known as eosinophilic leukemia, prompted by a chromosomal rearrangement, referred to as *FIP1L1-PDGFRA* leads to constitutive activation of the PDGFRA tyrosine domain, bringing patients to full remission within months of imatinib treatment [23, 24]. This chromosomal rearrangement is present in other PDGFRA related cancers as well [25]. Gene rearrangements involving *PDGFRB* have also been described, such as the one present in dermatofibrosarcoma protuberans a benign proliferative condition, in which the fusion *COL1A1-PDGFB* makes cells express more PDGFRB in a constitutive fashion [26]. Although glioblastoma therapy resistance is associated with the presence of autocrine PDGF-PDGFR loops, the use of specific inhibitors did not result in therapy improvement so far [27].

### ROS1, ALK, MET

Not all tyrosine kinase receptors have described physiological function and ligands, being sometimes referred to as orphan receptors. One such case is ROS1, which although almost unknown in relation to physiology is known to be upregulated or mutated in some tumors, especially NSCLC but not only [28]. ROS1 belongs to the insulin receptor superfamily as well as ALK and is structurally related to ALK and MET, what makes them share common inhibitors. MET is the receptor for the hepatocyte growth factor (HGF), shows normal low expression levels in most tissues and is aberrantly activated in solid tumors. A study revealed that NSCLC patients overexpressing MET present a disease free survival of 8 months while the lower expression correlates with a disease free survival of 53 months. MET is also overexpressed as a mechanism of resistance to EGFR positive tumors being treated with some TKIs. Common and non-specific MET inhibitors include cabozantinib, amuvatinib, crizotinib and foretinib, all of those also inhibiting other receptors such as ALK, AXL, VEGFR2, RET and KIT, which makes it difficult to evaluate the effect of MET inhibition as one never knows if only MET has been inhibited. More recently, a MET specific inhibitor has been developed, receiving the name tivantinib, which is still in clinical trials for NSCLC, colorectal, prostate and gastric tumors, showing some beneficial effects for the patients but its development still being questioned due to several adverse effects [29–32].

The search for therapy targets in tumors that still lack those is ongoing and a recent study just analyzed the possibility of using MET inhibitors for basal-like and triple-negative breast cancers, as the role of MET has been described in breast cancer development and these aggressive tumors lack other targets for approach. These are still pre-clinical studies but should be soon developed using human breast cancer samples for screening [33].

*ALK*, the acronym for anaplastic lymphoma kinase, is found to be rearranged in 3 to 13% of NSCLC and its inhibition mediated by TKIs is more effective than conventional chemotherapy alone. Its physiological role is related to brain embryogenesis, but fusion with other genes results in increased tyrosine kinase activity leading to tumor development through PLC, JAK-STAT, PI3K-AKT, mTOR, SHH, JUN-B, CRKL-C3G, RAP1, GTPase and MAPK cascades.

Crizotinib, a first generation tyrosine kinase inhibitor acts on ROS-1, MET and ALK, promoting longer progression free survival in NSCLC when compared to traditional chemotherapy [29].

Second generation ALK inhibitors, such as ceritinib, alectinib and brigatinib were developed mainly due to ALK+ tumor resistance, arising from *ALK* mutations C1156Y, L1196M, G1269A, F1174L, 1151Tins, L1152R, S1206Y, I1171T, G1202, D1203N and V1180L. ROS1 mutations such as G2032R also render tumors resistant to crizotinib [34]. Alternative pathway activation involving EGFR, KRAS, KIT, ERBB, MET and IGF-1R are also responsible for ALK+ tumor crizotinib resistance. Ceritinib promotes high response among those who failed responding to crizotinib and alectinib is ALK specific and circumvents L1196M resistance other than crossing blood-brain barrier treating brain metastasis, together with lorlatinib which is a third generation ALK inhibitor that also inhibits ROS1 and is effective against all known resistance mutants easily crossing blood-brain barrier [30, 35]. Several clinical trials are still ongoing to improve use of these ALK, MET, ROS1 tyrosine kinase inhibitors.

### RET

The single-pass transmembrane receptor tyrosine kinase called RET is required for the normal development of several cells and tissues, its dysregulation being present in some tumors. It is notable the role of this gene in the inherited cancer syndrome known as multiple endocrine neoplasia type 2. This syndrome is mostly characterized by the early occurrence of medullary thyroid carcinoma, possibly pheochromocytoma and other glands hyperplasia. RET has also been found as a fusion protein in a very small fraction of NSLCC patients. The common RET fusions found are *KIF5B-RET*, *CCDC6-RET*, *NCOA4-RET* and *TRIM33-RET* and they are not restricted to NSCLC, but can also be found in papillary thyroid carcinoma and myelonocytic leukemia [36, 37]. Cabozantinib and vandetanib are multikinase TKIs that have been approved by the FDA for the treatment of metastatic medullary thyroid carcinoma harboring RET alterations. Vandetanib also inhibits VEGFR and EGFR and RET resistance arises when the V804M mutation is present. Cabozantinib inhibits RET but also MET, VEGFR, AXL, KIT and FLT3 and is active in subsets of patients whose disease progressed during other TKIs treatment, including vandetanib [38].

A phase III clinical trial for cabozantinib in metastatic medullary thyroid carcinoma showed progression free survival of 11.2 months versus only 4.0 months in the placebo group. A similar phase III study using vandetanib showed progression free survival at 6 months in 83% of the patients comparing to 63% in the control group [39].

A recent phase II clinical trial tested erlotinib alone and cabozantinib alone or in combination with erlotinib to treat wild-type EGFR NSCLC patients in a randomized, controlled, open-label, multicenter study finding that progression free survival was improved in the cabozantinib arms. Although the status of *RET* mutations has not been described, cabozantinib being a pan-TKI with RET targeting depicts the possible contribution of this oncogene inhibition for the positive results [40].

### Vascular Endothelial Growth Factor Receptor (VEGFR)

What drives angiogenesis, such an important hallmark for so many cancer types has always been a key element for the development of specific therapy. Several molecules have been identified so far, but undoubtedly, VEGF family, including its tyrosine kinase receptor VEGFR is the most important one. The ligands can be of the A, B, C and D type, plus placental growth factor and they act on VEGFR, being described in subtypes 1, 2 and 3, all having specific physiologic and pathologic roles [41, 42].

Laboratory and clinical research have demonstrated so far that conditions such as hypoxia, inflammation, tumor suppressor inactivation and oncogene signaling all increase VEGF/VEGFR signaling [41, 42].

Several different approaches have been developed to inhibit VEGF signaling, including monoclonal neutralizing antibodies to circulating ligand, such as bevacizumab. VEGFR-2 blocking antibodies, such as ramucirumab, antibody-like decoy traps that bind both VEGF and placental growth factor, as aflibercept and several TKIs acting as specific inhibitors or pan-kinase inhibitors that also target VEGFR, namely sunitinib, sorafenib, pazopanib, axitinib, regorafenib, nintedanib, cabozantinib and vatalanib [41–43].

Clinical trials led to the approval of several of those inhibitors for specific tumors, but not all of the trials were successful, as for several tumors this pathway inhibition resulted in no quantifiable benefit regarding disease free progression or overall survival.

Among the successful trials we can cite the approval of bevacizumab for NSCLC and colorectal, ovarian and cervical cancers. Bevacizumab, ramucirumab and aflibercept have been approved as second line treatment for colorectal cancer while nintedanib and ramucirumab have been approved for second line treatment of NSCLC [42, 43].

TKIs were found to be mostly beneficial for other kinds of tumors, sunitinib, sorafenib, pazopanib, axitinib promoting improved disease free progression and overall survival for patients of renal cell carcinoma, as well as bevacizumab plus interferon does; sorafenib is approved for hepatocellular carcinoma, pazopanib for sarcomas and sunitinib for pancreatic neuroendocrine tumors [43].

Bevacizumab is a potent VEGFR inhibitor and finds use not only in cancer but other benign diseases in which angiogenesis plays a role, including inflammatory conditions and retinopathy [44]. Although for many years it was believed that the development of anti-angiogenesis agents would render tumors unviable, the early use of bevacizumab in glioblastoma multiforme tumors, one of the most aggressive central nervous system tumors known, showed that there is possibility of tumor resistance [45]. Tumors decrease the number of blood vessels due to bevacizumab therapy, surrounding edema is reduced, tomography images show rapid decrease in contrast enhancement, but several tumors still persist and progress, because the lack of vessels and hypoxic conditions promote HIF (hypoxia inducible factor) expression, upregulation of MET compensatory pathways and autocrine loops that maintain cells now adapted to rely on autophagy and keep alive. Different from other tyrosine kinase receptors that resist to TKIs through mutations that render them incapable of being bound by the inhibitors, VEGFR signaling pathways develop resistance through a much more complex and orchestrated mechanism that goes beyond mutation, HIF induction and all its signaling being pivotal, as well as the selection of non-VEGF dependent blood vessels and the metastatic growth of tumors in highly irrigated tissues such as liver, lungs and brain, which abrogates the need for neoangiogenesis [45–47].

### Others

**Fig. 1:**
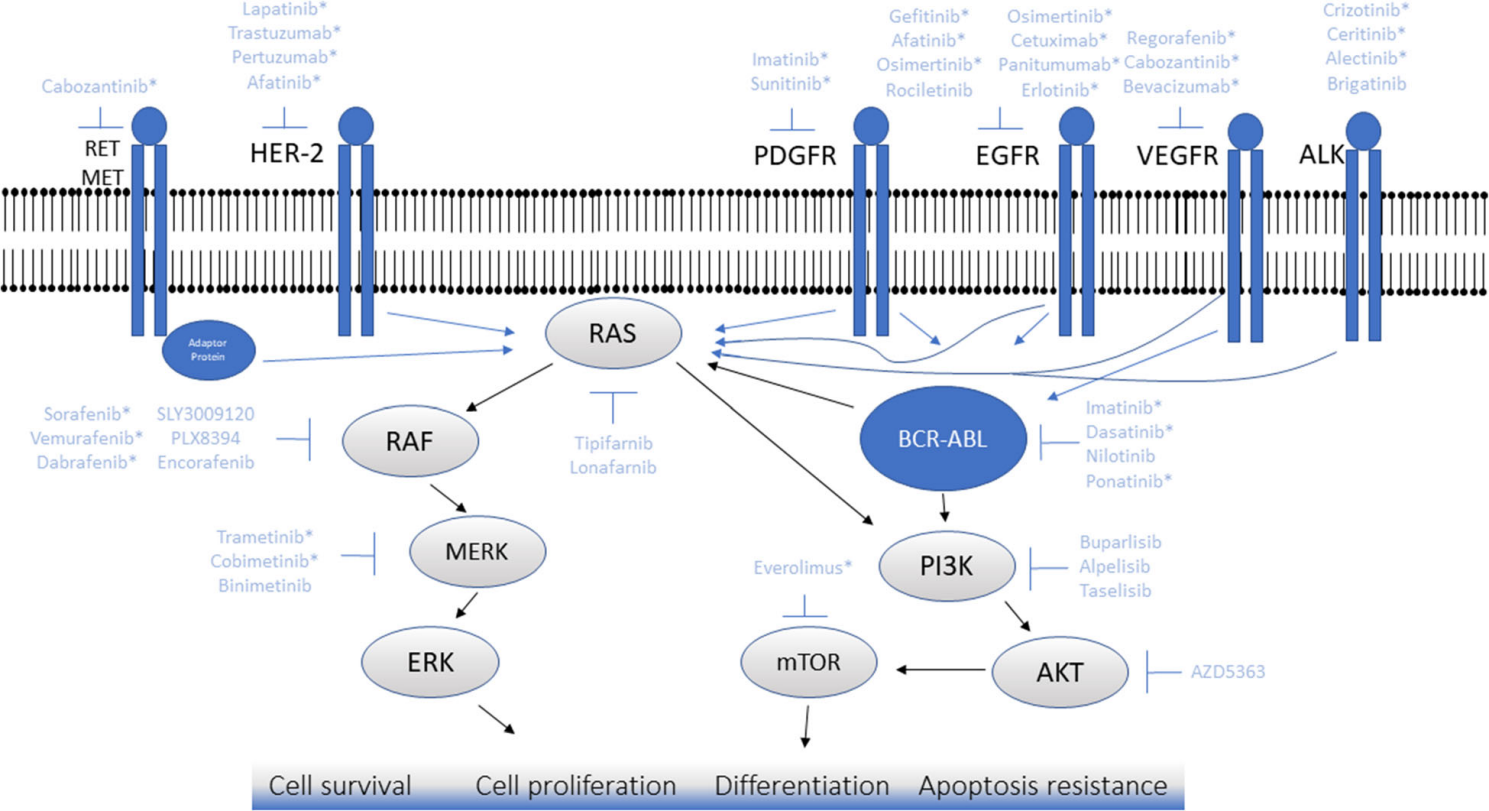
Examples of druggable targets and their inhibitors. Abnormal activation of receptors and downstream signaling pathways trigger cell survival, cell proliferation, differentiation and apoptosis resistance, contributing to tumorigenesis. Inhibitors are shown inhibiting their targets. FDA approved inhibitors*

There are still other important tyrosine kinase receptors involved in cell physiology and having a role in cancer development. Examples of such receptors are the fibroblast growth factor receptor FGFR and insulin-like growth factor receptors IGF-1, nonetheless, in spite of the fact that there are specific inhibitors to such receptors, uncountable clinical trials failed to show benefits or their use. Probably a lot more of their biology in normal cells and cancer cells has to be understood in order to design better inhibitor molecules and clinical trials [48–51]. Drugs being tested for tumors other than the FDA approved scenarios in the case of receptor tyrosine kinases are listed in table 1 and examples of inhibitors of each receptor can be seen in figure 1 (Table 1, Fig. 1).

**Table 1:** Examples of drugs targeting tyrosine kinases receptors that are being tested in different clinical trials

TARGET	DRUG	DISEASE	TRIAL PHASE	STATUS	CLINICAL TRIAL NUMBER
MET	Tivantinib	Advanced/Recurrent Gastric Cancer	II	Completed	NCT01152645
MET/EGFR/Topoisomerase	Tivantinib + cetuximab + irinotecan	Metastatic Colon Cancer	I/II	Completed	NCT01075048
MET/EGFR	Tivantinib + erlotinib	NSCLC	III	Completed	NCT01244191
MET/EGFR	Tivantinib + erlotinib	Locally Advanced or Metastatic NSCLC	II	Completed	NCT01395758
MET/Thymidylate Synthase	Tivantinib + FOLFOX	Advanced Solid Tumors and Previously Untreated Metastatic Adenocarcinoma of the Distal Esophagus, Gastroesophageal Junction or Stomach	I	Completed	NCT01611857
MET	Tivantinib	Metastatic Breast Cancer	II	Completed	NCT01575522
EGFR	Erlotinib	Bladder Cancer	II	Completed	NCT00380029
EGFR	Erlotinib	Advanced Head and Neck Cancer	II	Terminated	NCT00750555
EGFR	Gefitinib	Recurrent or Metastatic Esophageal or Gastroesophageal Junction Cancer	II	Completed	NCT00268346
EGFR	Gefitinib	Advanced or Metastatic Thyroid Cancer	II	Completed	NCT00095836
EGFR	Afatinib	Hormone-Refractory Prostate Cancer	II	Completed	NCT01320280
EGFR	Afatinib	Breast Cancer	II	Completed	NCT01325428
EGFR	Afatinib	Head and Neck Cancer	III	Terminated	NCT01345669
EGFR	Rociletinib or Erlotinib	Metastatic NSCLC	II	Terminated	NCT02186301
EGFR	Lapatinib + Ciscplatin + Radiotherapy	Head and Neck Cancer	II	Terminated	NCT00387127
PDGFR/Microtubule	Dasatinib + Ixabepilone	Metastatic Breast Cancer	I	Completed	NCT00924352
PDGFR/Microtubule	Dasatinib + Docetaxel	Metastatic Hormone-Refractory Prostate Cancer	II	Completed	NCT00439270
PDGFR/EGFR	Dasatinib + Erlotinib	NSCLC	II	Completed	NCT00826449
Multiple Receptor Tyrosine Kinases (PDGFR, c-KIT, VEGFRs)	Sunitinib	Pancreatic Neuroendocrine Tumors	III	Terminated	NCT00428597
Multiple Receptor Tyrosine Kinases (PDGFR, c-KIT, VEGFRs)	Sunitinib	Esophageal Cancer	II	Completed	NCT00702884
Multiple Receptor Tyrosine Kinases (PDGFR, c-KIT, VEGFRs)/Microtubule/EGFR	Sunitinib + Docetaxel + Trastuzumab	Advanced Breast Cancer	I	Completed	NCT00372424
ALK	Ceritinib	Tumors Characterized by Genetic Abnormalities in ALK	I	Completed	NCT01283516
MET and VEGFR	Cabozantinib	Prostate Cancer	II	Completed	NCT01834651
VEGFR/Microtubule	Bevacizumab + Paclitaxel	Metastatic Breast Cancer (HER-2 negative)	II	Active	NCT01663727

FOLFOX is a chemoterapy regimen (folinic acid, fluorouracil, oxaliplatin)

*NSCLC* Non-small Cell Lung Cancer, *ALK* Anaplastic Lymphoma Kinase

## Signalling pathways downstream receptor tyrosine kinases

### RAS network

Not only alterations in receptors are associated with malignant transformation and tumor progression, but abnormal activation is also observed in members of signaling pathways that are generally triggered by these tyrosine kinase receptors and regulate proliferation, cell survival, apoptosis, migration and cell differentiation. Numerous transduction signaling pathways have been dissected, which are activated in different tumors, and many target therapies have been developed; however, many challenges still need to be circumvented, among them the existence of crosstalks between the intracellular circuitry activated by these different receptors.

The membrane-bound small guanosine triphosphatases (GTPases) comprise a family of four members (HRAS, KRAS4A, KRAS4B and NRAS), that although related, have different functions. RAS proteins are found in two states: inactive when GDP bound and active when GTP bound. Activation of RAS recruits guanine nucleotide exchange factors (GEFs; e.g., SOS1) to the plasma membrane, promoting nucleotide exchange and formation of the RAS-GTP active form. GTPase-activating proteins (GAPs: e.g., neurofibrimin) induce the hydrolysis of RAS bound GTP, leading to the formation of inactive RAS-GDP [52]. Active RAS in turn triggers intracellular cascades of phosphorylation of downstream effectors, controlling energy metabolism, cell survival, proliferation, migration and invasion. In non-tumorigenic mammalian cells, the main and best studied RAS protein effectors are in the MAPK signaling pathway, comprising RAF/MEK/ERK and PI3K/AKT/mTOR transduction pathways. These mitogenic signaling cascades are hyperactivated in many neoplasias especially due to activating mutations [53].

Mutations in the three *RAS* genes have been described in more than 30% of human cancers and consist in the most common mutated oncogene family in neoplasias. *RAS* genes are mutated in different frequencies, *KRAS* being mutated in 85% of all RAS-driven cancers, *NRAS* in 12% and *HRAS* in 3% (COSMIC v82). *RAS* mutations are frequently found in pancreatic ductal adenocarcinoma (69–95%), colorectal adenocarcinoma (40–45%) and NSCLC (16–40%). However, in breast, melanoma, brain and ovarian tumors, these mutations are less common [54, 55]. All the mutations described result in high GTP loading, which in turn increases RAS activity, leading to uncontrolled cell proliferation, abnormal cell survival and apoptosis resistance, therefore, showing the involvement of *RAS* oncogene in malignant transformation and cancer development. In spite of the intensive research in this field, the development of effective drugs that inhibit *RAS* oncogenes has not been successful so far, because RAS isoforms have distinct properties and functions. Although the translocation and association of RAS proteins with plasma membrane is fundamental for its activation and to trigger downstream signaling pathways, the mechanisms that regulate these interactions among the isoforms through post-translational modifications and lipid processing are different. While HRAS is attached to the membrane by a farnesyltransferase catalyzed reaction, KRAS4B and NRAS undergo further modification by related geranylgeranyl isoprenoid formation [56]. Consequently, farnesyltransferase inhibitors (FTIs) were more efficient in preclinical studies comprising HRAS-driven cancers [57] and failed to demonstrate the same efficiency in tumors that harbor mutations in *KRAS* [58]. Tipifarnib and lonafarnib were the only FTI which advanced to Phase III clinical trials, but with poor clinical outcomes. The treatment with tipifarnib was evaluated in refractory advanced colon cancer, metastatic pancreatic cancer and advanced NSCLC, however it demonstrated minimal clinical activity and did not improve OS [59–61]. Tipifarnib was also tested in combination with gemcitabine, the standard chemotherapy agent used in advanced pancreatic cancers. Although the combination of gemcitabine and tipifarnib demonstrated antiproliferative activity in preclinical and in phases I and II clinical studies, the OS of patients was not increased when compared with the administration of gemcitabine as a single agent in phase III trials [62]. Lonafarnib was used in combination with paclitaxel and carboplatin in patients with metastatic, taxane-refractory/resistant NSCLC and the authors observed that the treatment was well tolerated and presented minimal toxicity, however without improving OS [63]. The failure in anti-RAS drug discovery decreased the studies in this field and promoted the development of alternative strategies to inhibit RAS activation. In the last years, a significant effort has been made to develop low-molecular-weight chemical inhibitors of the downstream effectors of RAS, notably the RAF-MEK-ERK and PI3K-AKT-mTOR signaling pathways and some of them have already been approved by the FDA while others are in different clinical trial phases. Although some of the downstream effectors of RAS are not tyrosine kinases, they are activated by such proteins, as is the case of EGFR, PDGFR and VEGFR, and because of that they are discussed in this article.

### RAF/MEK/ERK signaling pathway

The first kinase activated by RAS-GTP in the MAPK cascade is the serine/threonine-specific protein kinase RAF, comprising three tissue-specific isoforms: ARAF, BRAF and CRAF/RAF1. RAF activates MEK1 and MEK2 dual-specificity kinases, the only RAF known substrates, which in turn phosphorylate the effectors ERK1 and ERK2 related serine/threonine kinases. Activation of this signaling pathway culminates in the phosphorylation of cytoplasmic and nuclear targets regulating cell proliferation, survival, differentiation, apoptosis and in some circumstances negative feedback regulators of the RAF-MEK-ERK pathway [64]. Activation of RAF-MEK-ERK transduction circuit is sufficient to induce proliferation and migration of normal fibroblasts independent on upstream RAS signaling, reinforcing the participation of these effectors in cancer progression [65].

Mutations associated with RAF family are frequently associated with *BRAF* and even though *BRAF* mutations are genetic drivers in a wide range of tumors, they are mainly found in cancers that harbor *RAS* mutations, such as malignant melanoma, colorectal and thyroid carcinomas. Mutations in *BRAF* are found in up to 66% of melanoma patients, 18% of colorectal carcinomas and in 66% of papillary thyroid carcinoma cases and are associated with poor prognosis [66–69]. All the mutations are in the kinase domain, almost all of which are a single substitution of valine for glutamic acid at codon 600 (V600E) [66]. These mutations increase the kinase activity of BRAF and stimulate the phosphorylation of downstream effectors ERK1 and ERK2, increasing cell proliferation and survival and its identification provides new therapeutic opportunities [66]. On the other hand, mutations of *CRAF*, *ARAF* or *MEK1/2* are uncommonly described in human tumors [70]. However, in some lung cancer models that harbor *KRAS* mutations, CRAF is mediating oncogenic signaling from KRAS [71, 72], suggesting it would be a target for pharmacological inhibition. Moreover, as MEK is the only kinase that activates ERK and ERK is the only known substrate for MEK, the development of inhibitors for this signaling pathway is an attractive strategy in cancer therapy.

The participation of BRAF in tumor progression was reported in many studies. Overexpression of mutated *BRAF* into immortalized melanocytes induces anchorage-independent growth, mediates melanoma cell invasion and the development of tumors in mice [73–75]. On the other hand, inactivation of BRAF by RNA interference or small molecules leads to ERK phosphorylation inhibition, cell cycle arrest and apoptosis in preclinical models [76, 77] exclusively in BRAF-V600E-positive cells, indicating BRAF as a promising druggable target.

Sorafenib, the first RAF inhibitor developed, was designed to inhibit CRAF, but it also decreases the activity of wild-type BRAF and the oncogenic *BRAF* V600E mutant and is an antiangiogenic tyrosine kinase VEGFR/PDGFR-targeting drug. It was approved by the FDA in 2007 for advanced hepatocellular carcinoma, increasing OS and in 2013 for the treatment of locally recurrent or metastatic, progressive differentiated thyroid carcinoma refractory to radioactive iodine treatment. Although the treatment increases PFS, OS was not improved [78, 79]. Moreover, it has been reported that sorafenib treatment causes the development of skin lesions, including keratoses, keratocanthomas (KA) and squamous cell carcinomas (SCCs), suggesting that the molecule may not be efficient in RAS-driven tumors, since it induces a feedback activation of this signaling pathway, increasing proliferation of epithelial cells [79, 80].

Vemurafenib and dabrafenib, approved by the FDA in 2011 and 2013, respectively, improved OS and PFS of metastatic or unresectable melanoma patients when compared with dacarbazine and preferentially inhibit the V600E mutant form of *BRAF* over the wild-type form [81–84]. However, almost all patients relapsed due to development of drug resistance, in patients treated with vemufarenib the median time to progression being 7 months and with dabrafenib being 5 months [68]. This occurs due to the paradoxical activation of ERK signaling in tumor cells with wild-type *BRAF* or the ones that harbor *RAS* and *BRAF* mutations mutually [82]. Innumerous mechanisms have been proposed to explain the BRAF-target therapy acquired resistance, including increased PDGFR receptor tyrosine kinase-mediated activation of alternative oncogenic pathways, secondary mutations in *NRAS* [85], formation and transactivation of BRAF-CRAF heterodimers [86], upregulation of the *BCL2A1* anti-apoptotic gene [87], hyperactivation of CRAF driven by oncogenic *RAS* [86], increased migration capability conferred by CD271 overexpression [88] or activation of the other MAPKK COT [89]. Furthermore, *HRAS* mutations were detected in 60% of tumor samples from patients who developed KA and cutaneous SCCs after vemurafenib treatment [90].

Vemurafenib and dabrafenib were also evaluated in innumerous clinical trials for NSCLC and colorectal cancer, however, as a monotherapy it did not overcome the tumor strategies to progress. In lung adenocarcinomas, *BRAF* V600E mutant is found in only 1-2% of patients, conferring aggressiveness and resistance to currently available therapies including chemotherapy and radiotherapy [91]. Dabrafenib treatment could represent an option for patients with advanced NSCLC, but studies demonstrate only partial response. Moreover, such as in melanoma patients, drug resistance was observed and 30% of the treated group relapsed.

These studies reinforce the importance of identifying mutated genes and consequently activated signaling pathways in clinical practice and before administration of BRAF-target drugs improving patient’s response and avoiding side effects.

The observation that *RAS* oncogene overexpressed with BRAF^V600E^renders ERK signaling vemurafenib resistant and the essential participation of CRAF in lung cancers with mutations in *KRAS* leads to development of pan-RAF inhibitors, named LY3009120 and PLX8394, which do not activate MAPK signaling in tumors that harbor *RAS* mutations [92, 93]. These inhibitors block signals from RAF homo and heterodimers, including CRAF-containing dimers, therefore, overcoming paradoxical MAPK activation.

LY3009120 inhibited the proliferation of melanoma cells with either *BRAF* or NRAS and colorectal cancer cells with *BRAF* and *KRAS* mutations by inducing G0/G1 cell cycle arrest. Moreover, the treatment with LY3009120 inhibited the growth of melanoma cells that harbor *NRAS* mutations xenografts and KRAS-driven colorectal tumors *in vivo* [92, 94]. However, the continuous treatment of HCT 116 cells with LY3009120 leads to development of resistance as showed by the reactivation of RAF/MEK/ERK cascade, possibly by the crosstalking with AKT signaling pathway [94]. LY3009120 is in a phase I clinical trial for the treatment of advanced or metastatic melanoma, NSCLC and colorectal carcinomas (NCT02014116).

The other pan-RAF inhibitor developed, PLX8394, decreased the proliferation of vermurafenib resistant metastatic colorectal cancer cell lines by preventing RAF dimer formation and paradoxal MAPK signaling pathway activation [93, 95]. PLX8394 is being evaluated in phase I/IIa clinical trial for safety, pharmacokinetics and pharmacodynamics in patients with advanced *BRAF* mutated melanomas, thyroid carcinoma, colorectal cancer and NSCLC (NCT02428712).

Potent and highly selective allosteric MEK1/2 inhibitors were also developed for the treatment of oncogenic BRAF and RAS driven cancers and two of them, trametinib and cobimetinib, were approved as a single-agent therapy by the FDA for the treatment of V600E mutated metastatic melanoma [96, 97]. However, acquired resistance was developed within 6 to 7 months after treatment with trametinib monotherapy in nearly 50% of the patients, in part because of reprogramming of protein kinase network, leading to expression and activation of multiple RTKs, which in turn, stimulate the RAF-MERK-ERK pathway, circumventing MEK abrogation [98].

To overcome the development of resistance observed in patients treated with BRAF or MEK inhibitors as a single agent, it was believed that a more complete inhibition of the MAPK signaling pathway was required, so the combined therapy with trametinib and dabrafenib was approved by the FDA for the treatment of patients with *BRAF* V600E/K-mutant unresectable or metastatic melanoma in 2014.

The approval for the combination was based on results from an open-label phase I/II trial, which showed that trametinib combined with dabrafenib nearly doubled the duration of response and significantly improved ORR when compared with dabrafenib alone. The BRAF and MEK inhibitor combination was found to significantly reduce the incidence of secondary cutaneous squamous cell carcinoma. The approval of the agents in combination marks the first for a targeted therapy combination in advanced melanoma.

Uncountable phase III clinical trials, evaluating the combination of dabrafenib and trametinib in previously untreated melanoma patients with unresectable or metastatic disease harboring a *BRAF* V600E or V600K mutation, showed the improvement in PFS and OS compared with conventional chemotherapy or placebo, establishing the combined therapy as a standard treatment in melanoma harboring *BRAF* Val 600 mutations [99–101].

The decreased response to platinum-based chemotherapy and acquired resistance to vemurafenib and dabrafenib in patients with NSCLC harboring *BRAF* V600E mutations led to the development of a more effective targeted therapy combining dabrafenib and trametinib, which was approved by the FDA in 2015. That approval was based on results from a 3-cohort, multicenter, non-randomized, open-label study of patients with stage IV NSCLC. The combination of BRAF and MEK inhibitors demonstrated higher overall response and median PFS than dabrafenib monotherapy, establishing the combined therapy as a standard treatment in patients with advanced or metastatic NSCLC with *BRAF* V600E driver mutations. The safety profile was manageable, decreasing toxicity with thorough dose modification [102].

More recently, it has been shown that the combination of dabrafenib and trametinib treatment decreased ERK activation, cell proliferation and induced apoptosis in human cancer cell lines harboring non-V600 *BRAF* mutations, which accounts for approximately half of *BRAF*-mutated NSCLC [103]. This study shows evidences for the clinical use of these drugs for neoplasias harboring other *BRAF* mutations.

Another approach approved by the FDA for the treatment of metastatic melanomas with BRAF mutations is the combination of cobimetinib with vemurafenib. Cobimetinib is a highly specific selective, ATP-non-competitive inhibitor of MEK1/2 in neoplasias harboring *BRAF* V600E mutations. In human xenograft models, cobimetinib decreased tumor growth of colon and melanoma tumors containing *BRAF* mutations [104]. The combined therapy using cobimetinib and vemurafenib improved the median OS, PFS and the ORR in unresectable stage IIIC or stage IV melanoma patients harboring *BRAF* V600E mutations when compared with vemurafenib monotherapy [105, 106], demonstrating the clinical benefit of this treatment. Moreover, other MEK and BRAF inhibitors have been developed and several clinical trials are ongoing. Binimetinib is an allosteric selective, ATP-non-competitive inhibitor of MEK1/2 that demonstrated anti-tumoral activity by abrogating the growth of *NRAS*- and V600E *BRAF*-mutated melanomas in preclinical studies using in vitro and in vivo models [107]. In a non-randomized, open-label phase II study of advanced melanoma patients harboring *NRAS* or VAL600 *BRAF* mutations, binimetinib showed a partial response, providing the first target therapy to treat patients with *NRAS*-mutated melanomas [108]. Binimetinib has also been evaluated in combination with encorafenib, a highly selective BRAF inhibitor, in patients with advanced or metastatic melanoma with *BRAF* driver mutations. In this phase III clinical trial, the combined therapy with binimetinib plus encorafenib improved PFS and objective response rate by local and central review when compared with vemurafenib in *BRAF* mutant melanoma patients [109].

Furthermore, uncountable therapeutic strategies using MEK inhibitors in combination with other drugs to target tumors harboring *BRAF* and *RAS* mutations are under investigation. The efficiency of the combination of binimetinib and encorafenib plus cetuximab in the treatment of colorectal cancers harboring *BRAF* V600E mutations is in a phase III clinical development (NCT02928224). Biological evidence for the combination of binimetinib with erlotinib in the treatment of *KRAS* mutated NSCLC to overcome erlotinib acquired resistance was also evaluated, providing a personalized treatment based on the identification of signaling pathway dysregulations [110].

Network modeling analysis using Transcriptional Regulatory Associations in Pathways (TRAP) suggested CDK4 as an efficient target to be associated with MEK inhibitors in the treatment of melanoma harboring *NRAS* mutations which remains without effective therapy [111]. Cyclin-dependent kinases (CDKs) are a family of serine-threonine kinases that bind a regulatory protein called cyclin and the complex CDK-cyclin regulates the progression through the cell cycle, promoting cell proliferation. The complex cyclinD-CDK4 phosphorylates and inhibits members of the retinoblastoma (RB) protein family, including RB1, regulating the cell-cycle during G1/S transition. Biological and clinical evidence have showed that combination of ribociclib with MEK inhibitors as binimetinib or trametinib have increased anti-tumoral activity in neoplasias harboring *NRAS* mutations, including melanoma, NSCLC and colorectal carcinomas in preclinical models in vitro an in vivo [111–113].

Regarding the combination of BRAF and MEK inhibitors with immunomodulatory agents as pembrolizumab, durvalumab or atezolizumab, antibodies that target programmed cell death receptors (PD-1) or programmed cell death-ligand 1 (PD-L1), several trials are also in clinical development [114–118].

### PI3K/AKT/mTOR signaling pathway

Downstream to RAS there are the lipid kinases known as PI3Ks. These are heterodimeric proteins with one catalytic subunit of which there are three isoforms, each of them related to a specific gene: p110α/PIK3CA, p110β/PIK3CB, p110δ/PIK3CD, plus a regulatory subunit associated with cancer development by increasing cell survival, cell proliferation and conferring apoptosis resistance [35]. They phosphorylate phosphatidylinositol (4,5)-bisphosphate (PIP-2) to phosphatidylinositol (3,4,5)-triphosphate (PIP-3) on the plasma membrane, which in turn, recruits and activates phosphoinositide-dependent protein kinase 1 (PDK1). PDK1 phosphorylates the serine/threonine kinase at AKT/PKB Thr308 which then translocates to the plasma membrane, resulting in partial activation. AKT is completely activated upon its phosphorylation at Ser473 by mTOR complex 2 (mTORC2), a serine/threonine kinase, when it targets many proteins associated with cell survival or cell death depending on the cellular context, including mTORC1 [52]. PI3K pathway is negatively regulated by Phosphatase and Tensin Homolog (PTEN), which dephosphorylates PIP3, abrogating AKT activation. Innumerous genetic abnormalities associated with oncogenic transformation have been described in PI3K/AKT/mTOR pathway, including gain-of-function mutations and amplifications in *PIK3CA*, *AKT1* and *mTOR* oncogenes, and loss of function mutations, deletions or epigenetic inactivation in the tumor gene suppressor *PTEN* [52, 53]. Activating mutations in *PIK3CA* oncogene are found in around 30% of different tumors, including breast, colon, endometrium and prostate carcinomas [119]. *AKT1* mutations were described in breast, colorectal, ovarian and endometrial carcinomas and cause AKT1 constitutive activation [120]. The detailed knowledge of the PI3K/AKT/mTOR pathway leads to the development of several specific drugs some of which are currently in different phases of clinical trials.

Since PI3K/AKT signaling pathway is one of the mechanisms underlying hormonal therapy resistance in advanced breast carcinoma, PI3K inhibitors were used in combination with fulvestrant or tamoxifen. Buparlisib, an inhibitor of a pan-isorform class I PI3K, taken orally, increased PFS in association with fulvestrant in postmenopausal women with advanced or metastatic estrogen receptor (ER) positive HER-2 negative breast cancer harboring *PIK3CA* mutations in a phase III clinical trial [121, 122]. Buparlisib, is already being studied (phase IB) in association with lapatinib, a dual tyrosine kinase inhibitor which abrogates the HER-2/neu and EGFR pathways, in HER-2 positive advanced breast cancer that is resistant to trastuzumab, since the PI3K cascade is involved in trastuzumab resistance, and early conclusions demonstrate that this association is feasible for this kind of breast cancer [123].

When *PIK3CA* is mutated, the association of alpelisib, another alpha-specific PI3K inhibitor and fulvestrant showed good results in a phase I study of patients with advanced ER positive breast cancer on standard therapy [124]. There is a phase III study ongoing about the association of alpelisib or placebo with fulvestrant, and it aims to evaluate the PFS in two cohorts, one on mutated *PIK3CA* and the other with the wild type gene, and both stratified by the presence of lung and/or liver metastases, and prior CDK4/6 inhibitors treatment [122]. Other associations are being tested and in early phases of trials, as alpelisib and exemestane and letrozole, both antitumoral combinations, alpelisib and letrozole being tested for the safety and tolerability in patients with ER+ and HER-2 negative metastatic breast cancers that do not respond to endocrine therapy [122].

Another oral drug that is being studied in phase I is taselisib, a PI3K inhibitor with selectivity for the alpha isoform and preference for tumors that harbor *PIK3CA* mutations. The data showed that taselisib was effective on metastatic or locally advanced solid malignancies that progressed or failed standard therapy, showing antitumor activity at low doses [125]. When associated with other inhibitors such as fulvestrant, taselisib has demonstrated a higher antitumoral response in HER-2 negative and ER positive breast cancers with *PIK3CA* mutations if compared with the wild type [122].

PI3K/AKT signaling pathway is also hyperactivated in many B-cell malignancies being associated with tumor progression. A first-in-human phase IIa trial showed that copanlisib, a PI3K inhibitor with predominant inhibitory activity against both PI3K-α and PI3K-δ isoforms, has an antitumor effect as a single therapy in relapsed/refractory non-Hodkin’s lymphoma (NHL) and chronic lymphocytic leukemia [126]. Two phase III studies are in progress in indolent NHL and one additional Phase II study in diffuse large B-cell lymphoma (DLBCL), an aggressive subtype of NHL. The phase III clinical trials are randomized, double-blind, placebo-controlled study of copanlisib in rituximab refractory indolent NHL patients who have previously been treated with rituximab and alkylating agents (NCT02369016) or to evaluate the safety and efficacy of copanlisib plus rituximab versus rituximab single therapy in patients with relapsed NHL who have received at least one prior line of treatment, including rituximab and an alkylating agent (NCT02367040). The phase II is open-label, single arm study in patients with relapsed or refractory DLBCL to evaluate the efficacy and safety of copanlisib (NCT02391116). It is important to know that most of the tumors that were more affected by copanlisib had less activity of PTEN, and there was no association to *PIK3CA* mutation, despite the number of patients was not the best to conclude it definitely [127].

In metastatic castration resistant prostate cancer (mCRPC), it was shown that AKT1 activation induces resistance to docetaxel and prednisolone chemotherapy [128]. Preclinical studies demonstrated the antitumoral activity of AZD5363, a pan-AKT inhibitor, as a monotherapy. Moreover, the combination of AZD5363 with hormonal therapy improved efficacy of PI3K/AKT-targeted treatment in PTEN-negative prostate carcinoma models, implicating this pharmacological strategy in this type of cancer [129]. There is an ongoing phase I/II trial in mCRPC that evaluates the association of AZD5363 with androgen receptor antagonist enzalutamide (NCT02525068). There are many studies about combination of AZD5363 with other drugs to potentialize its effect [130–132], but just a few clinical trials, which means that there is a long way to FDA approved treatments involving AKT inhibition when it is super activated.

mTOR inhibitors are also being studied, and they seem to be a good treatment option for some kinds of cancers, including gynecological ones, since their use alone or in combination with other hormonal drugs are good strategies that need further studies [133]. An example is the everolimus, an mTOR inhibitor approved by the FDA for the treatment of many types of cancer, including kidney cancer and some neuroendocrine tumors. Association of everolimus with endocrine therapy showed a good option for HER-2- and ER+ metastatic breast cancer [134]. In renal cell carcinoma it was observed that everolimus associated with other drugs, as levantinib, cabozantinib and nivolumab, has a better antitumoral effect than everolimus alone [135].

All these evidences show that altered PI3K/AKT/mTOR altered pathway may induce tumorigenesis, and treatments that focus on these mutations and dysfunctions are targets of further studies, moreover, association of drugs can interrupt tumor progression in more than one point and avoid resistance caused by pathway crosstalk.

### ABL1 kinase

The *ABL1 (*Abelson murine leukemia viral oncogene homolog 1) proto-oncogene encodes tyrosine kinases that can be found both in the cytoplasm and the nucleus of different cell types and that shuttle between the two compartments. Activation of ABL1 is mediated by different receptor tyrosine kinases, including EGFR, PDGFR and VEGFR [136]. Furthermore, ABL is also activated by intracellular signals such as DNA damage and oxidative stress, leading to p73 phosphorylation and apoptosis induction [137]. Activated ABL1 phosphorylates a large number of substrates, such as adaptors, other kinases, cytoskeletal proteins, transcription factors and chromatin modifiers, which in turn, activate innumerous signaling pathways, including RAS/RAF/MEK, PI3K/AKT and lipids and protein phosphatases, thereby regulating cell differentiation, cell proliferation, cell survival, cell migration, cell invasion and stress response [138]. BCR-ABL1 is associated with the increased expression of cytokines as granulocyte colony-stimulating factor and granulocyte-macrophage colony-stimulating factor (GM-CSF) [139].

Oncogenic activation of the ABL1 kinase is induced as a consequence of the t(9;22)(q34;q11) chromosome translocation in Philadelphia-positive human leukemia, generating the new fusion gene *BCR-ABL1*, a cytoplasmic-target tyrosine kinase with constitutive activity, leading to abnormal cell proliferation and increased resistance to apoptosis [136]. The presence of the BCR-ABL1 protein is a genetic hallmark of CML, characterized by the neoplastic transformation of haematopoietic stem cells. The requirement of BCR-ABL1 to the development of CML, renders ABL1 an attractive pharmacological target. In 2001, FDA approved imatinib, as the first-line treatment for Philadelphia chromosome-positive CML, both in adults and children. Imatinib is a potent inhibitor of the tyrosine kinases ABL, ARG, PDGFR and KIT, inducing apoptosis of BCR-ABL positive cells [140]. The FDA has also approved imatinib for use in adults with relapsed or refractory Philadelphia chromosome-positive acute lymphoblastic leukemia (Ph + ALL) [141]. It was reported that imatinib induced complete cytogenetic response as analyzed by in situ hybridization in more than 80% of the patients newly diagnosed with CML in chronic phase (CP), however, in patients with more advanced phases, the complete remission was less frequent [142]. Acquired resistance to imatinib was observed in 40% to 60% of the patients since BCR-ABL positive cells persists after the target therapy and one of the mechanisms described was the emergence of point mutations in the kinase domain of *BCR-ABL* gene that prevent drug interaction [142]. More than 90 different mutations have been described in *BCR-ABL* gene, conferring variable degrees of resistance to imatinib treatment.

Dasatinib, another BCR-ABL and also a Src family tyrosine kinase inhibitor was approved by the FDA as an important strategy for the treatment of patients with newly diagnosed chronic-phase CML and for imatinib-resistant or -intolerant patients with CP or advanced-phase CML or Ph + ALL [143].

Nilotinib was also developed and approved by the FDA in 2007 for the treatment of adult patients with newly diagnosed Ph + CML-CP and patients with imatinib-resistant or imatinib-intolerant Ph + CML in CP or accelerated phase (AP). Nilotinib is a selective BCR-ABL kinase inhibitor, structurally related to imatinib and exhibited 10–30 fold more potency than imatinib in inhibiting BCR-ABL tyrosine kinase activity and proliferation of BCR-ABL expressing cells. It was showed that treatment with nilotinib is more effective because it induces less diverse *BCR-ABL* mutations than imatinib in patients with chronic myeloid leukemia in CP, however, the incidence of the T315I mutation was similar with nilotinib and imatinib. Moreover, the progression to accelerated phase/blast crisis was lower with nilotinib than imatinib in patients with emergent *BCR-ABL* mutations [144].

More recently, ponatinib was developed and approved by the FDA in 2016 to treat patients with Ph + CML and Ph + ALL carrying T315I mutation, which was resistant to imatinib or nilotinib [145]. Ponatinib was designed applying ARIAD’s computational and structure-based drug design platform to inhibit the kinase activity of BCR-ABL protein with more potency and specificity. Ponatinib was designed to target the mutated BCR-ABL isoforms that render leukemia cells resistant to treatment with existing tyrosine kinase inhibitors, especially including the T315I mutation for which no effective therapy exists [146]. Drugs being tested for tumors other than the FDA approved scenarios in the case of signalling pathways downstream molecules are listed in table 2 and examples of inhibitors of each downstream molecule can be seen in figure 1 (Table 2, Fig. 1).

**Table 2:** Examples of drugs targeting downstream effectors of tyrosine kinase receptors that are in clinical development

TARGET	DRUG	DISEASE	TRIAL PHASE	STATUS	CLINICAL TRIAL NUMBER
BRAF;CRAF/RTK	Sorafenib + Vinorelbine	Breast Cancer	I/II	Completed	NCT00828074
BRAF;CRAF/Microtubule/DNA replication	Sorafenib + Paclitaxel + Carboplatin	Ovarian Cancer	II	Completed	NCT00390611
Pan RAF	LY3009120	Advanced or Metastatic Cancer	I	Active	NCT02014116
Pan RAF	PLX8394	Advanced Cancers	I/II	Terminated	NCT02012231
Pan RAF	PLX8394	Advanced Unresectable Solid Tumors	I/II	Recruiting	NCT02428712
MEK1/2	Trametinib	Oral Cavity Squamous Cell Cancer	II	Completed	NCT01553851
MEK1/2	Trametinib	Cancer	II	Active	NCT01072175
MEK1/2	Dabrafenib	Papillary Thyroid Carcinoma	Unknown	Completed	NCT01534897
MEK1/2/BRAF/BRAF	Binimetinib + Encorafenib + Vemurafenib	Melanoma	III	Active	NCT01909453
MEK1/2/BRAF/EGFR	Binimetinib + Encorafenib + Cetuximab	Metastatic Colorectal Cancer	III	Recruiting	NCT02928224
MEK1/2/CDK4/6	Binimetinib + Palbociclib	NSCLC	I/II	Recruiting	NCT03170206
BRAF/EGFR	Encorafenib + Cetuximab	Metastatic Colorectal Cancer	I/II	Active	NCT01719380
BRAF	Encorafenib	Melanoma and Metastatic Colorectal Cancer	I	Active	NCT01436656
BRAF + PD-1/PD-L1 axis	Vemurafenib + Pembrolizumab	Melanoma	I	Recruiting	NCT02818023
BRAF + PD-1/PD-L1 axis	Vemurafenib + Atezolizumab	Melanoma		Active	NCT01656642
PI3K/Estrogen Receptor	Buparsilib (BKM120) + Fulvestrant	Metastatic Breast Cancer	III	Active	NCT01633060
PI3K/EGFR, HER2neu	Buparsilib (BKM120) + Lapatinib	Breast Cancer	I/II	Suspended (data analysis)	NCT01589861
PI3K	Alpelisib + Fulvestrant	Breast Cancer	III	Recruiting	NCT02437318
PI3K	Alpesilib + Fulvestrant	Breast Cancer	II	Not yet recruiting	NCT03386162
PI3K/Aromatase	Alpelisib + Fulvestrant + Letrozole	Breast Cancer	II	Recruiting	NCT03056755
PI3K/mTOR/Aromatase	Alpelisib + Everolimus + Exemestane	Breast Cancer, Kidney Cancer, Pancreatic Neuroendocrine Cancer	I	Active	NCT02077933
PI3K	Taselisib + Fulvestrant	Breast Cancer	III	Active	NCT02340221
PI3K/Aromatase	Taselisib + Letrozole	Breast Cancer	II	Completed	NCT02273973
PI3K	Taselisib	Recurrent Squamous Cell Lung Carcinoma, Stage IV Squamous Cell Lung Carcinoma	II	Active	NCT02785913
PI3K	Copanlisib (BAY80-6946)	Non-Hodgkin Lymphoma	III	Active	NCT02369016
PI3K/CD20	Copanlisib (BAY80-6946) + Rituximab	Non-Hodgkin Lymphoma	III	Recruiting	NCT02367040
PI3K	Copanlisib (BAY80-6946)	Diffuse Large B-cell Lymphoma (DLBCL)	II	Active	NCT02391116
AKT/Androgen Receptor	AZD5363 + Enzalutamide	Adenocarcinoma of the Prostate	II	Recruiting	NCT02525068
AKT/Microtubule	AZD5363 + Paclitaxel	Advanced Gastric Cancer	II	Recruiting	NCT02451956
AKT	AZD5363	Metastatic Castrate-Resistant Prostate Cancer (mCRPC)	I	Completed	NCT01692262

*NSCLC* Non-Small Cell Lung Cancer, *RTK* receptor tyrosine kinase

## Conclusion

We are a few years from the great breakthrough of testing and approving imatinib in the late nineties and early 2001, the “magic bullet” for treating cancer, opening up the gates and calling all the attention to the new era of cancer treatment at the time thinking we would fully transition from classical chemotherapy to target therapy alone. Nowadays, an equilibrium has been reached as classical chemotherapy is still in use and in combination with target therapy, but the number of molecules that have been developed the same way as imatinib is easily reaching the hundreds, some of which are in the market and some of which failed at some point during development, but they all undoubtedly led us to deeply understand cell proliferation in cancer and non-cancer states, especially when resistance arose and had to be circumvented. The molecular characterization of tumors and the use of specific drugs targeting specific defects in single patients is the closest we got to personalized medicine and accompanying that we improved rates of overall survival, progression free survival, disease free survival and other markers. Nowadays a lot has been developed, although not enough and there is a clear notion of the path that has to be followed to develop more of these specific inhibitors while clinical practice and evidence is bringing more and more knowledge on the proper use of the currently available arsenal proposing and testing drug combinations and regimens or searching classical targets in tumors not known to harbor them.

## Abbreviations

ABL1: Abelson murine leukemia viral oncogene homolog 1
ALK: Anaplastic lymphoma kinase
ALL: Acute lymphoblastic leukemia
AP: Accelerated phase
CDKs: Cyclin-dependent kinases
CML: Chronic myelogenous leukemia
CP: Chronic phase
DLBCL: Diffuse large B-cell lymphoma
EGFR: Epidermal growth factor receptor
ER: Estrogen receptor
FDA: Food and drug administration
FGFR: Fibroblast growth factor receptor
FTIs: Farnesyltransferase inhibitors
GAPs: GTPase-activating proteins
GEFs: Guanine nucleotide exchange factors
GIST: Gastrointestinal stromal tumors
GM-CSF: Granulocyte-macrophage colony-stimulating factor
HER-2: Human epidermal growth factor receptor 2
HGF: Hepatocyte growth factor
HIF: Hypoxia inducible factor
IGF-1: Insulin-like growth factor receptors
KA: Keratocanthomas
mCRPC: Metastatic castration resistant prostate cancer
mTOR: Mammalian target of rapamycin
mTORC1: mTOR complex 1
mTORC2: mTOR complex 2
NHL: Non-Hodkin’s lymphoma
NSCLC: Non-small cell lung carcinoma
ORR: Overall response rate
OS: Overall survival
PDGFR: Platelet-derived growth factor receptor
PDK-1: Phosphoinositide-dependent protein kinase 1
PFS: Progression-free survival
Ph +: Philadelphia-positive
PIP-2: Phosphatidylinositol (4,5)-bisphosphate
PIP-3: Phosphatidylinositol (3,4,5)-triphosphate
PTEN: Phosphatase and Tensin Homolog
RB: Retinoblastoma
RTKs: Receptor tyrosine kinases
SCCs: Squamous cell carcinomas
SCLC: Small cell lung cancer
TCGA: The cancer genome atlas
TKIs: Tyrosine kinase inhibitors
TRAPs: Transcriptional regulatory associations in pathways
VEGF: Vascular endothelial growth factor
VEGFR: Vascular endothelial growth factor receptor
